# Investigating the Role of Cytomegalovirus as a Cause of Stillbirths and Child Deaths in Low- and Middle-Income Countries Through Postmortem Minimally Invasive Tissue Sampling

**DOI:** 10.1093/cid/ciaf098

**Published:** 2025-03-10

**Authors:** Sithembiso Velaphi, Zachary J Madewell, Beth Tippett-Barr, Dianna M Blau, Emily A Rogena, Sanjay G Lala, Sana Mahtab, Peter J Swart, Victor Akelo, Dickens Onyango, Kephas Otieno, Joyce A Were, Quique Bassat, Carla Carrilho, Inacio Mandomando, David Torres-Fernandez, Rosauro Varo, Ronita Luke, Francis Moses, Philip Nwajiobi-Princewill, Ikechukwu Udo Ogbuanu, Julius Ojulong, Shams El Arifeen, Emily S Gurley, Nega Assefa, Letta Gedefa, Lola Madrid, J Anthony G Scott, Henok Wale, Jane Juma, Adama Mamby Keita, Karen L Kotloff, Samba O Sow, Milagritos D Tapia, Portia Mutevedzi, Cynthia G Whitney, Shabir A Madhi

**Affiliations:** Department of Paediatrics, Faculty of Health Sciences, University of the Witwatersrand, Johannesburg, South Africa; Global Health Center, US Centers for Disease Control and Prevention, Atlanta, Georgia, USA; Nyanja Health Research Institute, Salima, Malawi; Global Health Center, US Centers for Disease Control and Prevention, Atlanta, Georgia, USA; Department of Pathology, University of Nairobi, Nairobi, Kenya; Department of Paediatrics and Child Health, Faculty of Health Sciences, University of the Witwatersrand, Johannesburg, South Africa; South African Medical Research Council Vaccines and Infectious Diseases Analytics Research Unit, Faculty of Health Sciences, University of the Witwatersrand, Johannesburg, South Africa; Division of Anatomical Pathology, National Health Laboratory Services and University of the Witwatersrand, Johannesburg, South Africa; Centers for Disease Control and Prevention–Kenya, Kisumu, Kenya; Kisumu County Department of Health, Kisumu, Kenya; Center for Global Health Research, Kenya Medical Research Institute, Kisumu, Kenya; Center for Global Health Research, Kenya Medical Research Institute, Kisumu, Kenya; ISGlobal–Hospital Clínic, Universitat de Barcelona, Barcelona, Spain; Centro de Investigação em Saúde de Manhiça, Maputo, Mozambique; ICREA, Barcelona, Spain; Pediatrics Department, Hospital Sant Joan de Déu, Universitat de Barcelona, Barcelona, Spain; CIBER de Epidemiología y Salud Pública, Instituto de Salud Carlos III, Madrid, Spain; Department of Pathology, Faculty of Medicine, Eduardo Mondlane University, Maputo, Mozambique; Department of Pathology, Maputo Central Hospital, Maputo, Mozambique; ISGlobal–Hospital Clínic, Universitat de Barcelona, Barcelona, Spain; Centro de Investigação em Saúde de Manhiça, Maputo, Mozambique; Instituto Nacional de Saúde, Ministério de Saúde, Maputo, Mozambique; ISGlobal–Hospital Clínic, Universitat de Barcelona, Barcelona, Spain; ISGlobal–Hospital Clínic, Universitat de Barcelona, Barcelona, Spain; Centro de Investigação em Saúde de Manhiça, Maputo, Mozambique; Ministry of Health and Sanitation, Freetown, Sierra Leone; Ministry of Health and Sanitation, Freetown, Sierra Leone; Department of Medical Microbiology, National Hospital Abuja, Abuja, Nigeria; Crown Agents, Freetown, Sierra Leone; ICAP, Columbia University, Makeni, Sierra Leone; International Center for Diarrhoeal Disease Research, Dhaka, Bangladesh; International Center for Diarrhoeal Disease Research, Dhaka, Bangladesh; Department of Epidemiology, Johns Hopkins Bloomberg School of Public Health, Baltimore, Maryland, USA; College of Health and Medical Sciences, Haramaya University, Harar, Ethiopia; Department of Infectious Disease Epidemiology, London School of Hygiene and Tropical Medicine, London, United Kingdom; College of Health and Medical Sciences, Haramaya University, Harar, Ethiopia; College of Health and Medical Sciences, Haramaya University, Harar, Ethiopia; Department of Infectious Disease Epidemiology, London School of Hygiene and Tropical Medicine, London, United Kingdom; Department of Infectious Disease Epidemiology, London School of Hygiene and Tropical Medicine, London, United Kingdom; College of Health and Medical Sciences, Haramaya University, Harar, Ethiopia; Centre pour le Développement des Vaccins, Ministère de la Santé, Bamako, Mali; Centre pour le Développement des Vaccins, Ministère de la Santé, Bamako, Mali; Department of Pediatrics and Department of Medicine, Center for Vaccine Development and Global Health, University of Maryland School of Medicine, Baltimore, Maryland, USA; Centre pour le Développement des Vaccins, Ministère de la Santé, Bamako, Mali; Department of Pediatrics and Department of Medicine, Center for Vaccine Development and Global Health, University of Maryland School of Medicine, Baltimore, Maryland, USA; Emory Global Health Institute, Emory University, Atlanta, Georgia, USA; Emory Global Health Institute, Emory University, Atlanta, Georgia, USA; South African Medical Research Council Vaccines and Infectious Diseases Analytics Research Unit, Faculty of Health Sciences, University of the Witwatersrand, Johannesburg, South Africa; Wits Infectious Diseases and Oncology Research Institute, Faculty of Health Sciences, University of the Witwatersrand, Johannesburg, South Africa

## Abstract

**Background:**

There is paucity of information on the role of cytomegalovirus (CMV) infection as a cause of stillbirths or childhood deaths in low- and middle-income countries (LMICs). We investigated attribution of CMV disease in the causal pathway to stillbirths and deaths in children <5 years of age in 7 LMICs participating in the Child Health and Mortality Prevention Surveillance (CHAMPS) network.

**Methods:**

We analyzed stillbirths and decedents enrolled between December 2016 and July 2023. Deaths were investigated using postmortem minimally invasive tissue sampling with histopathology and molecular diagnostic investigations of tissues and body fluids, along with review of clinical records. Multidisciplinary expert panels reviewed findings and reported on the causal pathway to death.

**Results:**

CMV was detected in 19.5% (1140/5841) of all evaluated deaths, including 5.0% (111/2204), 6.2% (139/2229), 41.2% (107/260), 68.1% (323/474), and 68.2% (460/674) of stillbirths, neonates (deaths <28 days postnatal), early infants (28 to <90 days), late infants (90 to <365 days), and children (12 to <60 months), respectively. CMV disease was attributed in the causal pathway to death in 0.9% (20/2204) of stillbirths, 0.8% (17/2229) of neonates, 13.1% (34/260) of early infants, 9.7% (46/474) of late infants, and 3.3% (22/674) of children. Decedents with CMV disease, compared with those without CMV disease in the causal pathway, were more likely to have severe microcephaly (38.2% vs 21.1%; adjusted odds ratio [aOR], 2.2 [95% confidence interval {CI}, 1.3–3.6]) and to have human immunodeficiency virus (HIV) (36.9% vs 6.2%; aOR, 10.9 [95% CI, 6.5–18.5]).

**Conclusions:**

CMV disease is an important contributor to deaths during infancy and childhood and is often associated with severe microcephaly and HIV infection. Improving management of CMV in children with HIV and a vaccine to prevent CMV are needed interventions.

## Teaser

Postmortem investigation in 7 low- and middle-income countries identified cytomegalovirus (CMV) disease as an important contributor to childhood deaths, particularly those with severe microcephaly and HIV infection. Further research on maternal vaccination and empiric CMV management in HIV-infected children is needed.

Cytomegalovirus (CMV) infection is common in children [1, 2], with approximately one-third of infections acquired peripartum, 40%–65% through breastfeeding, and the remainder via other modes [3–5]. While most in utero infections are asymptomatic, CMV can cause stillbirths and severe illness in newborns [6, 7]. Postnatal CMV infection is typically asymptomatic but can lead to severe sepsis-like syndrome in preterm infants [8]. CMV establishes latency in the host with the potential for periodic reactivation, particularly in immunocompromised individuals. In high-income countries, congenital CMV (cCMV) accounts for 3%–10% of deaths in symptomatic infants and 0.3%–1.0% of all infants with cCMV [9, 10]. Children with hospital diagnoses of cCMV have been reported to have 18.4 times odds of dying by 5 years of age relative to matched controls [11]. However, information on CMV's role in stillbirths and deaths among children under 5 in low- and middle-income countries (LMICs) is limited.

In LMICs, determining the cause of death in children often relies on limited clinical assessments, with or without verbal autopsy. Postmortem blood or tissue sampling is rare [12]. Consequently, pathogen-specific causes of death are often imputed, rather than being based on biological confirmation [13, 14]. Postmortem minimally invasive tissue sampling (MITS) combined with histopathology and molecular pathogen detection offers more detailed insight into the causes of death, particularly for infectious diseases [14–17]. These granular data are essential for prioritizing preventive strategies and improving treatment of severely ill children. This study analyzed CMV's contribution to stillbirths and under-5 child deaths, identified through mortality surveillance in 7 countries in Africa and Asia. We examined CMV infection and disease prevalence, assessed its role in the causal pathway leading to death, and described associated conditions in CMV-related stillbirths and child deaths.

## METHODS

Child Health and Mortality Prevention Surveillance (CHAMPS) methods for identifying deaths and determining their causes among children aged <5 years and stillbirths have been previously described [18–20]. CHAMPS, a multisite study across 6 African countries (Ethiopia, Kenya, Mali, Mozambique, Sierra Leone, and South Africa) and 1 South Asian country (Bangladesh), evaluates causes of deaths using postmortem MITS on eligible stillbirths and deceased children <5 years of age. Characteristics of the study sites have previously been described by Salzberg et al [18]. Collected tissue and fluid samples undergo comprehensive microbiological, molecular, and histopathological testing [18, 21]. CHAMPS also gathers additional data through standardized verbal autopsies using the World Health Organization's (WHO) 2016 instrument, clinical record abstraction (if records are available), postmortem anthropometric measurements, and photography.

### Sample Collection and Analysis

When a stillbirth or death in a child <5 years of age living within the site-specific catchment area is reported, CHAMPS staff seeks informed consent from parents/guardians for enrollment. Deaths reported within 24 hours (or 72 hours with refrigeration) are eligible for MITS. MITS is undertaken in a standardized manner under sterile conditions. Biopsy needles are used to collect tissues samples from the lungs, brain, and liver in all children and placenta in stillbirths. Additionally, blood, cerebrospinal fluid (CSF), stool, and nasopharyngeal secretions are collected.

Site laboratories perform routine microbiological cultures on blood and CSF specimens, along with assays for human immunodeficiency virus (HIV), *Mycobacterium tuberculosis*, and malaria. For molecular pathogen detection, 4 TaqMan Array Cards (TACs; Thermo Fisher Scientific, Waltham, Massachusetts) targeting 116 pathogens are used on lung tissue, blood or CSF, rectal swabs, and nasopharyngeal swabs [22]. The TAC panels for lung tissue, blood/CSF, and nasopharyngeal specimens include probes for identifying CMV. Histopathological analyses of brain, liver, and lung tissues are conducted at site laboratories using standard stains. If indicated by TAC results or initial pathology findings, immunohistochemistry for specific pathogens, including CMV, is conducted at the United States Centers for Disease Control and Prevention.

### Determining Cause of Death

A Determination of Cause of Death (DeCoDe) panel, comprised of pediatricians, neonatologists, obstetricians, epidemiologists, pathologists, and/or microbiologists, was established at each site [23, 24]. The DeCoDe process was standardized across sites through training and use of diagnostic standards [23]. Panels reviewed case findings, determined the sequence of events leading to death, and assigned diagnoses using WHO's *International Classification of Diseases, 10th Revision* (*ICD-10*) for deaths among children aged ≥28 days, and *International Classification of Diseases for Perinatal Mortality* (*ICD-PM*) for deaths in the perinatal/neonatal period. The DeCoDe panels categorized CMV disease as (1) underlying cause if CMV disease initiated the fatal sequence, (2) antecedent cause if it played a role in the chain of events, or (3) immediate cause if it directly led to death [25, 26].

CMV infection was defined as CMV detection on the TAC assay in nasopharyngeal swabs, lung tissue, CSF, or blood samples. CMV disease was defined as the presence of histopathologic changes (intranuclear viral inclusions, positive immunohistochemistry, pneumonitis, hepatitis, encephalitis, meningitis) on any of lung, liver, or brain samples; or when CMV polymerase chain reaction (PCR) reactivity was present on 2 or more specimen types (with nasopharyngeal and lung tissues being considered as a single site). Cases identified in stillbirths or neonates within 14 days of birth were classified as cCMV [27]. DeCoDe panels determine if CMV contributed to the death pathway, considering coexisting conditions and CMV disease severity.

### Data Management and Analysis

Frequency distributions were presented for all decedents, categorized into groups without CMV infection or disease, with CMV infection, and with CMV disease anywhere in the causal pathway. Analysis was stratified by age group: stillbirths, neonates (deaths <28 days after birth), early infants (28 to <90 days), late infants (90 to <365 days), and children (12 to <60 months). Proportions of deaths with CMV in the causal pathway with reactive CMV PCR from 2 sites (blood, CSF, lung specimens, or nasopharyngeal/oropharyngeal swabs) or abnormal histopathological changes on any lung, liver, or brain specimen were reported. Histopathology findings for kidney and heart were included for the few cases where these organs were inadvertently sampled during MITS. TAC PCR cycle threshold (Ct) values were analyzed for decedents with CMV disease in the causal pathway, CMV disease present but not in the causal pathway, and CMV infection without histological evidence of disease. Anthropometric characteristics (weight-for-age, height-for-age, head circumference-for-age, and weight-for-height Z-scores), comorbidities, and coinfections within the causal pathway were evaluated by age group, including decedents with CMV infection where CMV was not attributed as a cause and those with nonreactive CMV PCR results.

We used logistic regression to evaluate unadjusted and adjusted associations between various characteristics (age group, sex, location of death, site, HIV status, head circumference, malnutrition as causal or other significant condition) and the presence or absence of CMV in the causal pathway to death. This analysis excluded stillbirths and neonatal cases due to potentially different pathophysiologic mechanisms of CMV infection in this group. As a sensitivity analysis, we performed another logistic regression excluding deaths with CMV infection from the reference category. Odds ratios (ORs) and 95% confidence intervals (CIs) were reported, with *P* < .05 considered statistically significant. All analyses were performed using R software, version 4.3.1 (R Foundation for Statistical Computing, Vienna, Austria).

### Ethics Considerations

The ethics committees at each site and at Emory University, Atlanta, Georgia, USA, approved the overall protocol and site ethics committees approved site-specific protocols, respectively. The MITS procedure was undertaken once written informed consent was obtained from the deceased's parent/guardian.

## RESULTS

### Burden of CMV Infections and Disease by Age and Country

Of 5841 decedents enrolled from the 7 CHAMPS sites with DeCoDe results available, 2204 (37.7%) were stillbirths, 2229 (38.2%) neonates (<28 days), 260 (4.5%) early infants (28 to <90 days), 474 (8.1%) late infants (90 to <365 days), and 674 (11.5%) children (12 to <60 months). Overall, 19.5% (n = 1140) had CMV infection, including 5.0% (111/2204), 6.2% (139/2229), 41.2% (107/260), 68.1% (323/474), and 68.2% (460/674) of stillbirths, neonates, early infants, late infants, and children, respectively. CMV disease was diagnosed in 7.7% (450/5841) of decedents. Nevertheless, CMV disease was only attributed by the DeCoDe panel in the causal pathway to death in 2.4% (139/5941) of decedents (Table 1), including in 0.9% (20/2204), 0.8% (17/2229), 13.1% (34/260), 9.7% (46/474), and 3.3% (22/674) of stillbirths, neonates, early infants, late infants, and children, respectively. Among decedents with CMV disease in the causal pathway, CMV was attributed as underlying, antecedent, and immediate causes in 54 (38.8%), 50 (36.0%), and 59 (42.4%) cases, respectively. In decedents with CMV attributed in the causal pathway to death, it was the underlying cause in all 20 (100%) stillbirths and 64.7% (11/17) of neonatal deaths, but less so in other age groups: 29.4% (10/34), 15.2% (7/46), and 27.3% (6/22) of early infants, late infants, and children, respectively. In 22 decedents, an initial localized CMV infection (eg, pneumonitis) was diagnosed, followed by disseminated CMV infection, resulting in CMV being listed more than once in the chain of events leading to death. Six of 34 (17.6%) early-infant, 1 of 46 (2.2%) late-infant, and 1 of 22 (4.5%) childhood decedents had been diagnosed with cCMV antemortem. Sixteen of the 17 (94.1%) neonatal deaths with CMV in the causal chain had cCMV.

**Table 1.** Frequencies of Cytomegalovirus Infection and Disease Among All Deaths Across All Sites, by Age Group, Child Health and Mortality Prevention Surveillance (CHAMPS) Network, December 2016–July 2023

Characteristic	All Deaths (n = 5841)	Stillbirth (n = 2204)	Neonate (<28 d) (n = 2229)	Early Infant (28 to <90 d) (n = 260)	Late Infant (90 to <365 d) (n = 474)	Child (12 to <60 mo) (n = 674)
CMV infection^a^	1140 (19.5)	111 (5.0)	139 (6.2)	107 (41.2)	323 (68.1)	460 (68.2)
CMV disease^b^	450 (7.7)	41 (1.9)	48 (2.2)	61 (23.5)	151 (31.9)	149 (22.1)
Abnormal histopathology	130 (28.9)	11 (26.8)	12 (25.0)	36 (59.0)	48 (31.8)	23 (15.4)
Positive CMV PCR in ≥2 specimens	372 (82.7)	35 (85.4)	42 (87.5)	38 (62.3)	123 (81.5)	134 (89.9)
CMV disease in the causal pathway^c^	139 (2.4)	20 (0.9)	17 (0.8)	34 (13.1)^d^	46 (9.7)^d^	22 (3.3)^d^
Abnormal histopathology	100 (71.9)	8 (40.0)	11 (64.7)	31 (91.2)	38 (82.6)	12 (54.5)
Positive CMV PCR in ≥2 specimens	78 (56.1)	16 (80.0)	13 (76.5)	14 (41.2)	23 (50.0)	12 (54.5)
Immediate cause	59 (42.4)	0 (0.0)	1 (5.9)	22 (64.7)	27 (58.7)	9 (40.9)
Antecedent cause	50 (36.0)	0 (0.0)	6 (35.3)	17 (50.0)	17 (37.0)	10 (45.5)
Underlying cause	54 (38.8)	20 (100)	11 (64.7)	10 (58.8)	7 (15.2)	6 (27.3)

Data are presented as No. (%).

Abbreviations: CMV, cytomegalovirus; PCR, polymerase chain reaction.

^a^CMV infection: TaqMan Array Card PCR positive on any specimen.

^b^CMV disease: presence of histopathologic changes on any specimen OR disseminated CMV, defined as positive CMV PCR in ≥2 specimens (considering nasopharyngeal/oropharyngeal swabs and lung tissues as 1 specimen).

^c^CMV in the causal pathway: CMV is the immediate, antecedent, or underlying cause of death. Twenty-two deaths had CMV multiple times in the causal chain, including 14 with CMV as immediate and antecedent cause, 4 with CMV as underlying and antecedent cause, 2 with CMV as immediate and underlying cause, and 2 with CMV as immediate, antecedent, and underlying cause.

^d^Six early-infant deaths, 1 late-infant death, and 1 child death with CMV disease in the causal pathway were previously diagnosed as having congenital CMV before death.

**Figure 1.**
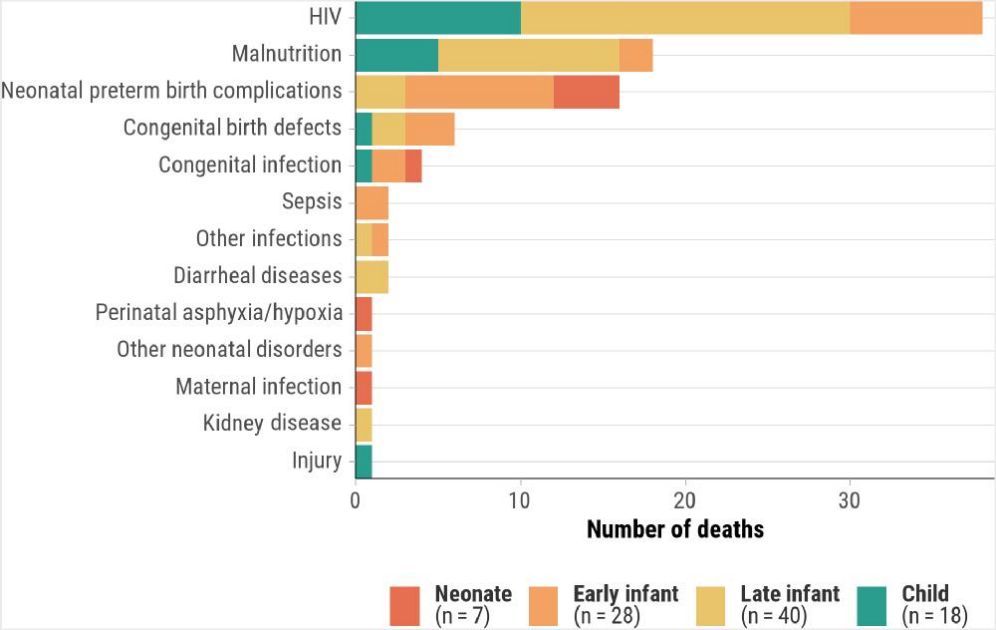
Underlying causes of death when cytomegalovirus is the immediate or antecedent cause, by age group, Child Health and Mortality Prevention Surveillance (CHAMPS) network, December 2016–July 2023. Abbreviation: HIV, human immunodeficiency virus.

**Figure 2.**
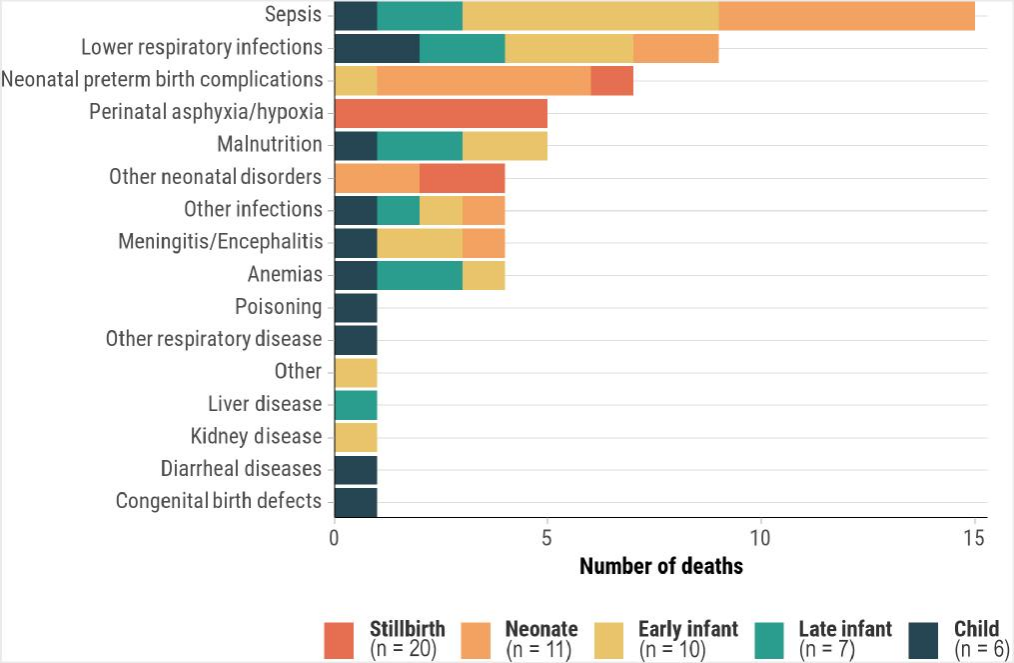
Immediate or antecedent causes of death when cytomegalovirus is the underlying cause, by age group, Child Health and Mortality Prevention Surveillance (CHAMPS) network, December 2016–July 2023.

CMV infection prevalence in stillbirths and deaths in children aged <5 years was lower in Bangladesh (3.0%) and Ethiopia (6.2%) compared to other African countries (14.5%–21.8%) (Supplementary Table 1). Similarly, deaths with CMV disease were lower in Bangladesh (1.0%) and Ethiopia (2.7%), compared to other African countries (4.9%–16.4%). In countries with higher proportions of deaths with CMV infection, it was more prevalent in late infants (50%–80.6%) and children (46.7%–76.9%). However, CMV disease in the causal pathway was more common in early infants (2.9%–18.5%) and late infants (7.8%–15.0%) than in children (1.9%–5.0%). In decedents where CMV disease was an immediate or antecedent cause of death, frequent underlying causes were HIV among late infants (50.0% [20/40]) and children (55.6% [10/18]), preterm birth–related complications among neonates (57.1% [4/7]), and preterm birth–related complications (32.1% [9/28]) and HIV (28.6% [8/28]) among early infants (Figure 1). For deaths where CMV was the underlying cause, immediate causes included intrauterine hypoxia/asphyxia in stillbirths (25.0% [5/20]); sepsis (54.5% [6/11]) and preterm birth–related complications (45.5% [5/11]) among neonates (<28 days); and sepsis (39.1% [9/23]), lower respiratory infections (30.4% [7/23]), and malnutrition (21.7% [5/23]) in other age groups, with sepsis more prevalent in early infants (60.0% [6/10]) (Figure 2).

### Diagnostics and Histopathology of CMV in the Causal Chain

Among 139 decedents with CMV disease in the causal pathway, 93.3% (126/135) had reactive TAC PCR on lung samples, 88.3% (91/103) in nasopharyngeal swabs, 87.4% (76/87) in blood, and 60.3% (47/78) in CSF. Among decedents with PCR available from multiple sites, 29.9% (41/137) were positive on 3 specimens (lung or nasopharyngeal/oropharyngeal swab, blood, CSF) and 27.0% (37/137) on 2 specimens (Table 2). Complete sample sets were available for 54.0% (75/139) of CMV deaths. Among deaths with CMV disease in the causal chain, median Ct values in lung (26.0 vs 30.7), nasopharyngeal swabs (26.0 vs 29.2), blood (29.9 vs 32.4), and CSF (31.0 vs 33.7) were lower than those without CMV disease in the causal pathway (*P* values <.001) (Supplementary Table 2).

**Table 2.** Positive Reverse-Transcription Polymerase Chain Reaction and Abnormal Histopathology Findings Among Deaths With Cytomegalovirus Disease in the Causal Pathway, by Age Group, Child Health and Mortality Prevention Surveillance (CHAMPS) Network, December 2016–July 2023

Specimen	All Deaths With CMV in Causal Pathway (n = 139)	Stillbirth (n = 20)	Neonate (<28 d) (n = 17)	Early Infant (28 to <90 d) (n = 34)	Late Infant (90 to <365 d) (n = 46)	Child (12 to <60 mo) (n = 22)
Positive CMV RT-PCR						
Any specimen	137/139 (98.6)	20/20 (100)	17/17 (100)	34/34 (100)	44/46 (95.7)	22/22 (100)
Blood	76/87 (87.4)	16/18 (88.9)	14/16 (87.5)	13/14 (92.9)	21/24 (87.5)	12/15 (80.0)
CSF	47/78 (60.3)	10/11 (90.9)	12/15 (80.0)	12/14 (85.7)	11/23 (47.8)	2/15 (13.3)
Lung	126/135 (93.3)	20/20 (100)	13/16 (81.2)	33/34 (97.1)	42/45 (93.3)	18/20 (90.0)
NP/OP swab	91/103 (88.3)	5/7 (71.4)	9/11 (81.8)	23/25 (92.0)	36/39 (92.3)	18/21 (85.7)
Abnormal histopathology						
Any specimen	100/139 (71.9)	8/20 (40.0)	11/17 (64.7)	31/34 (91.2)	38/46 (82.6)	12/22 (54.5)
Lung	98/139 (70.5)	7/20 (35.0)	11/17 (64.7)	31/34 (91.2)	37/46 (80.4)	12/22 (54.5)
Liver	36/135 (26.7)	2/16 (12.5)	4/17 (23.5)	20/34 (58.8)	10/46 (21.7)	0/22 (0.0)
Brain	20/124 (16.1)	3/14 (21.4)	1/17 (5.9)	10/34 (29.4)	5/40 (12.5)	1/19 (5.3)
Heart	4/52 (7.7)	0/4 (0.0)	0/7 (0.0)	2/11 (18.2)	2/19 (10.5)	0/11 (0.0)
Kidney	5/28 (17.9)	0/4 (0.0)	0/2 (0.0)	4/10 (40.0)	1/8 (12.5)	0/4 (0.0)
Types of abnormal histopathology						
Lung						
Viral inclusions	61/139 (43.9)	5/20 (25.0)	6/17 (35.3)	26/34 (76.5)	21/46 (45.7)	3/22 (13.6)
Immunohistochemistry	74/139 (53.2)	6/20 (30.0)	7/17 (41.2)	26/34 (76.5)	27/46 (58.7)	8/22 (36.4)
Viral pneumonitis	92/139 (66.2)	2/20 (10.0)	10/17 (58.8)	31/34 (91.2)	37/46 (80.4)	12/22 (54.5)
Liver						
Viral inclusions	21/135 (15.6)	1/16 (6.2)	1/17 (5.9)	13/34 (38.2)	6/46 (13.0)	0/22 (0.0)
Immunohistochemistry	31/135 (23.0)	2/16 (12.5)	3/17 (17.6)	17/34 (50.0)	9/46 (19.6)	0/22 (0.0)
Viral hepatitis	24/135 (17.8)	1/16 (6.2)	3/17 (17.6)	16/34 (47.1)	4/46 (8.7)	0/22 (0.0)
Brain						
Viral inclusions	5/124 (4.0)	0/14 (0.0)	1/17 (5.9)	3/34 (8.8)	1/40 (2.5)	0/19 (0.0)
Immunohistochemistry	7/124 (5.6)	1/14 (7.1)	0/17 (0.0)	4/34 (11.8)	2/40 (5.0)	0/19 (0.0)
Meningitis/encephalitis	10/124 (8.1)	1/14 (7.1)	1/17 (5.9)	6/34 (17.6)	2/40 (5.0)	0/19 (0.0)
Intracranial calcifications	12/124 (9.7)	3/14 (21.4)	0/17 (0.0)	4/34 (11.8)	4/40 (10.0)	1/19 (5.3)
Heart						
Viral inclusions	1/52 (1.9)	0/4 (0.0)	0/7 (0.0)	1/11 (9.1)	0/19 (0.0)	0/11 (0.0)
Immunohistochemistry	2/52 (3.8)	0/4 (0.0)	0/7 (0.0)	0/11 (0.0)	2/19 (10.5)	0/11 (0.0)
Myocarditis	3/52 (5.8)	0/4 (0.0)	0/7 (0.0)	2/11 (18.2)	1/19 (5.3)	0/11 (0.0)
Kidney						
Viral inclusions	3/28 (10.7)	0/4 (0.0)	0/2 (0.0)	2/10 (20.0)	1/8 (12.5)	0/4 (0.0)
Immunohistochemistry	4/28 (14.3)	0/4 (0.0)	0/2 (0.0)	3/10 (30.0)	1/8 (12.5)	0/4 (0.0)
Nephritis	2/28 (7.1)	0/4 (0.0)	0/2 (0.0)	2/10 (20.0)	0/8 (0.0)	0/4 (0.0)

Data are presented as no./No. (%).

Abbreviations: CMV, cytomegalovirus; CSF, cerebrospinal fluid; NP/OP, nasopharyngeal/oropharyngeal; RT-PCR, reverse-transcription polymerase chain reaction.

**Figure 3.**
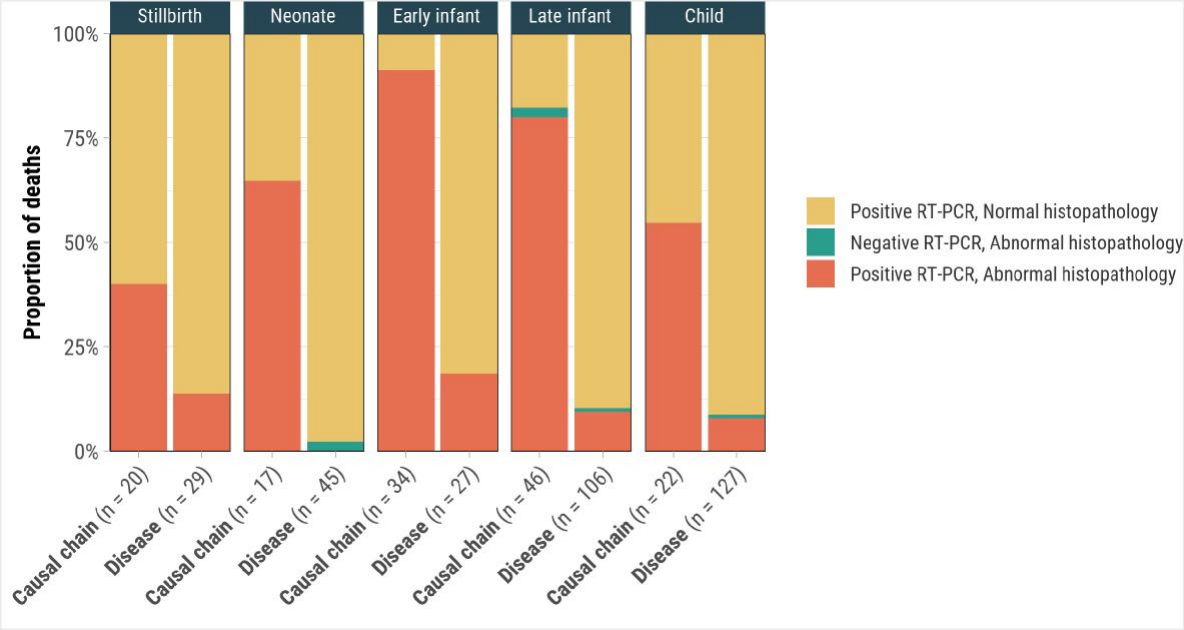
Cytomegalovirus (CMV) reverse-transcription polymerase chain reaction (RT-PCR) and CMV-associated abnormal histopathology findings for deaths with CMV in the causal chain (n = 139) and CMV disease but not in the causal chain (n = 311), by age group, Child Health and Mortality Prevention Surveillance (CHAMPS) network, December 2016–July 2023.

Histopathology revealed evidence of CMV disease in 71.9% (100/139) of cases with CMV attributed in the causal pathway (Table 2), primarily in lung specimens with viral pneumonitis (66.2% [92/139]), positive immunohistochemical staining (53.2% [74/139]), and viral inclusion bodies (43.9% [61/139]). Liver and brain abnormalities were found in 26.7% (36/135) and 16.1% (20/124) of cases, respectively. Early infants showed the highest proportion of changes, with lung abnormalities in 91.2% (31/34) and liver abnormalities in 58.8% (20/34), compared to 40.0% (8/20) in stillbirths and 64.7% (11/17) in neonates. One stillbirth with CMV in the causal chain had placental abnormalities, including chorioamnionitis with scattered CMV inclusions. Among the 100 decedents with CMV disease in the causal pathway and histopathological features of CMV disease, 44.0% (n = 44) had abnormalities in 2 or more organs and 56.0% (n = 56) in 1 organ. Histopathologic abnormalities were more frequent among deaths with CMV in the causal chain (ranging from 40.0% in stillbirths to 91.2% in early infants) compared to cases where CMV disease was present but not considered causal by DeCoDe panels (0% in neonates to 18.5% in early infants) across age groups (Figure 3).

### Characteristics of Deaths With CMV in the Causal Pathway

Of 139 deaths with CMV disease in the causal chain, 57.6% were male, 86.3% (120/139) died in a healthcare facility, 28.1% (39/139) were HIV infected, and 20.1% (28/139) were HIV exposed but uninfected (Table 3). HIV infection prevalence was highest in late infants (43.5% [20/46]) and children (45.5% [10/22]). Excluding 38 deaths with HIV as the underlying cause, 65.8% (25/38) of late-infant and child CMV deaths were severely underweight, 28.9% (11/38) were severely stunted, and 51.4% (19/37) were severely wasted. Severe microcephaly was present in 76.5% of neonates, 58.8% of early infants, and less frequently in late infants (32.6%) and children (18.2%). Excluding decedents with HIV as the underlying cause, the proportions of CMV-attributed deaths that were severely underweight and severely wasted were 92.3% (12/13) and 83.3% (10/12) in Kenya, 60.0% (3/5) and 0% (0/5) in Mozambique, 56.2% (9/16) and 60.0% (9/15) in Sierra Leone, and 80.8% (21/26) and 43.8% (7/16) in South Africa, respectively (Supplementary Table 3).

**Table 3.** Characteristics of Deaths With Cytomegalovirus Disease in Causal Pathway, by Age Group, Child Health and Mortality Prevention Surveillance (CHAMPS) Network, December 2016–July 2023

Characteristic	All Deaths With CMV in Causal Pathway (n = 139)	Stillbirth (n = 20)	Neonate (<28 d) (n = 17)	Early Infant (28 to <90 d) (n = 34)	Late Infant (90 to <365 d) (n = 46)	Child (12 to <60 mo) (n = 22)
Sex						
Female	59 (42.4)	10 (50.0)	9 (52.9)	14 (41.2)	19 (41.3)	7 (31.8)
Male	80 (57.6)	10 (50.0)	8 (47.1)	20 (58.8)	27 (58.7)	15 (68.2)
Location of death						
Facility	120 (86.3)	18 (90.0)	17 (100.0)	29 (85.3)	38 (82.6)	18 (81.8)
Community	19 (13.7)	2 (10.0)	0 (0.0)	5 (14.7)	8 (17.4)	4 (18.2)
Hospital length of stay, h, median (IQR) (n = 71)	67 (18–425)	…	53 (12–113)	777 (36–1238)	56 (20–178)	72 (42–274)
Site						
Bangladesh	7 (5.0)	4 (20.0)	2 (11.8)	1 (2.9)	0 (0.0)	0 (0.0)
Ethiopia	5 (3.6)	2 (10.0)	0 (0.0)	1 (2.9)	0 (0.0)	2 (9.1)
Kenya	24 (17.3)	1 (5.0)	2 (11.8)	7 (20.6)	11 (23.9)	3 (13.6)
Mali	5 (3.6)	0 (0.0)	0 (0.0)	1 (2.9)	3 (6.5)	1 (4.5)
Mozambique	22 (15.8)	7 (35.0)	1 (5.9)	1 (2.9)	6 (13.0)	7 (31.8)
Sierra Leone	22 (15.8)	0 (0.0)	3 (17.6)	3 (8.8)	9 (19.6)	7 (31.8)
South Africa	54 (38.8)	6 (30.0)	9 (52.9)	20 (58.8)	17 (37.0)	2 (9.1)
Time from death to MITS, h, median (IQR) (n = 101)	14 (4–26)	8 (3–29)	17 (3–34)	19 (7–45)	15 (12–23)	6 (2–10)
HIV status						
Uninfected or unknown	72 (51.8)	12 (60.0)	9 (52.9)	22 (64.7)	17 (37.0)	12 (54.5)
Exposed but uninfected	28 (20.1)	8 (40.0)	7 (41.2)	4 (11.8)	9 (19.6)	0 (0.0)
Infected	39 (28.1)	0 (0.0)	1 (5.9)	8 (23.5)	20 (43.5)	10 (45.5)
Weight-for-age Z-score (n = 38)^a^						
Normal (≥2 SD)	8 (21.1)	…	…	…	5 (19.2)	3 (25.0)
Moderate underweight (<−2 to −3 SD)	5 (13.2)	…	…	…	3 (11.5)	2 (16.7)
Severe underweight (<−3 SD)	25 (65.8)	…	…	…	18 (69.2)	7 (58.3)
Height-for-age Z-score (n = 38)^a^						
Normal (≥2 SD)	17 (44.7)	…	…	…	11 (42.3)	6 (50.0)
Moderate stunting (<−2 to −3 SD)	10 (26.3)	…	…	…	7 (26.9)	3 (25.0)
Severe stunting (<−3 SD)	11 (28.9)	…	…	…	8 (30.8)	3 (25.0)
Weight-for-height Z-score (n = 37)^a^						
Normal (≥2 SD)	9 (24.3)	…	…	…	5 (20.0)	4 (33.3)
Moderate wasting (<−2 to −3 SD)	9 (24.3)	…	…	…	6 (24.0)	3 (25.0)
Severe wasting (<−3 SD)	19 (51.4)	…	…	…	14 (56.0)	5 (41.7)
Mid-upper arm circumference (cm) Z-score (n = 38)^a^						
Normal (≥2 SD)	15 (39.5)	…	…	…	8 (30.8)	7 (58.3)
Moderate malnutrition (<−2 to −3 SD)	5 (13.2)	…	…	…	4 (15.4)	1 (8.3)
Severe malnutrition (<−3 SD)	18 (47.4)	…	…	…	14 (53.8)	4 (33.3)
Head circumference Z-score						
Normal (≥2 SD)	48 (40.3)	…	2 (11.8)	8 (23.5)	23 (50.0)	15 (68.2)
Moderate microcephaly (<−2 to −3 SD)	19 (16.0)	…	2 (11.8)	6 (17.6)	8 (17.4)	3 (13.6)
Severe microcephaly (<−3 SD)	52 (43.7)	…	13 (76.5)	20 (58.8)	15 (32.6)	4 (18.2)
Birth weight						
Extremely low birth weight	9 (6.5)	2 (10.0)	6 (35.3)	1 (2.9)	0 (0.0)	0 (0.0)
Very low birth weight	14 (10.1)	3 (15.0)	4 (23.5)	6 (17.6)	1 (2.2)	0 (0.0)
Low birth weight	24 (17.3)	8 (40.0)	2 (11.8)	7 (20.6)	7 (15.2)	0 (0.0)
Normal weight	34 (24.5)	4 (20.0)	4 (23.5)	5 (14.7)	18 (39.1)	3 (13.6)
Missing	58 (41.7)	3 (15.0)	1 (5.9)	15 (44.1)	20 (43.5)	19 (86.4)
Count of causal conditions identified						
1	24 (17.3)	14 (70.0)	4 (23.5)	2 (5.9)	2 (4.3)	2 (9.1)
2	33 (23.7)	4 (20.0)	2 (11.8)	9 (26.5)	13 (28.3)	5 (22.7)
3	30 (21.6)	2 (10.0)	5 (29.4)	9 (26.5)	9 (19.6)	5 (22.7)
≥4	52 (37.4)	0 (0.0)	6 (35.3)	14 (41.2)	22 (47.8)	10 (45.5)
Median (IQR)	3 (2–4)	1 (1–2)	3 (2–4)	3 (2–4)	3 (2–4)	3 (2–4)
Deemed preventable or preventable under certain conditions from DeCoDe panel	101 (72.7)	7 (35.0)	12 (70.6)	25 (73.5)	38 (82.6)	19 (86.4)

Data are presented as No. (%) unless otherwise indicated.

Abbreviations: CMV, cytomegalovirus; DeCoDe, Determination of Cause of Death panel; HIV, human immunodeficiency virus; IQR, interquartile range; MITS, minimally invasive tissue sampling; SD, standard deviation.

^a^Excludes deaths with HIV as underlying cause.

### Other Pathogens Isolated in Deaths With CMV Disease in the Causal Pathway

Among 139 deaths with CMV in the causal chain, frequent bacterial coinfections in the causal pathway were *Klebsiella pneumoniae* (31.7% [44/139]), *Streptococcus pneumoniae* (13.7% [19/139]), *Acinetobacter baumannii* (10.8% [15/139]), and *Staphylococcus aureus* (7.9% [11/139]). Frequent viral, fungal, and parasitic coinfections in the causal pathway were HIV (27.3% [38/139]), *Pneumocystis jirovecii* (14.4% [20/139]), and *Plasmodium falciparum* (5.8% [8/139]), respectively (Supplementary Figure 1).

### Comparing Deaths With CMV in the Causal Pathway With Deaths With Negative CMV Diagnostic Tests

Adjusting for variables shown in Table 4, the odds of CMV disease in the causal pathway were higher for HIV-infected cases (adjusted OR [aOR], 10.94 [95% CI, 6.52–18.47]) with HIV uninfected as reference; early infants (aOR, 4.30 [95% CI, 2.27–8.30]) or late infants (aOR, 3.48 [95% CI, 2.00–6.24]) with children as reference; died in a healthcare facility (aOR, 2.00 [95% CI, 1.13–3.75]) with community as reference; or had severe microcephaly (aOR, 2.18 [95% CI, 1.31–3.62]) (Table 4). Excluding cases with CMV infection or CMV disease from the reference group, the odds of CMV in the causal chain were higher for decedents with HIV infection (aOR, 35.07 [95% CI, 16.49–80.51]), late infants (aOR, 2.75 [95% CI, 1.43–5.45]), and decedents with severe microcephaly (aOR, 2.42 [95% CI, 1.31–4.52]) (Supplementary Table 4).

**Table 4.** Associations Between Characteristics and Cytomegalovirus Disease in the Causal Pathway Among Deaths in Infants and Children Aged 28 Days to <5 Years, Child Health and Mortality Prevention Surveillance (CHAMPS) Network, December 2016–July 2023 (N = 1408)

Characteristic	CMV in Causal Pathway (n = 102)	CMV Not in Causal Pathway (n = 1306)	Crude OR (95% CI)	*P* Value	Adjusted^a^ OR (95% CI)	*P* Value
Age group				<.001		<.001
Early infant	34 (33.3)	226 (17.3)	4.46 (2.57–7.88)		4.30 (2.27–8.30)	
Late infant	46 (45.1)	428 (32.8)	3.19 (1.91–5.46)		3.48 (2.00–6.24)	
Child	22 (21.6)	652 (49.9)	Reference		Reference	
Sex				.227		.134
Male	62 (60.8)	713 (54.6)	1.29 (.86–1.96)		1.41 (.90–2.22)	
Female	40 (39.2)	592 (45.4)	Reference		Reference	
Location of death				.005		.017
Facility	85 (83.3)	922 (70.9)	2.06 (1.24–3.62)		2.00 (1.13–3.75)	
Community	17 (16.7)	379 (29.1)	Reference		Reference	
Site				.034		.528
Bangladesh	1 (1.0)	9 (0.7)	0.82 (.04–4.53)		0.99 (.05–6.08)	
Ethiopia	3 (2.9)	73 (5.6)	0.30 (.07–.86)		0.56 (.12–1.80)	
Kenya	21 (20.6)	309 (23.7)	0.50 (.28–.86)		0.73 (.38–1.38)	
Mali	5 (4.9)	84 (6.4)	0.44 (.15–1.05)		0.58 (.18–1.53)	
Mozambique	14 (13.7)	228 (17.5)	0.45 (.23–.83)		0.48 (.23–.97)	
Sierra Leone	19 (18.6)	316 (24.2)	0.44 (.25–.77)		0.63 (.32–1.18)	
South Africa	39 (38.2)	287 (22.0)	Reference		Reference	
HIV status				<.001		<.001
Infected	38 (36.9)	90 (6.2)	8.12 (5.12–12.77)		10.94 (6.52–18.47)	
Uninfected	65 (63.1)	1365 (93.8)	Reference		Reference	
Head circumference Z-score				<.001		.011
Severe microcephaly (<−3 SD)	39 (38.2)	276 (21.1)	2.66 (1.70–4.16)		2.18 (1.31–3.62)	
Moderate microcephaly (<−2 to −3 SD)	17 (16.7)	163 (12.5)	1.97 (1.07–3.45)		1.43 (.73–2.70)	
Normal (≥2 SD)	46 (45.1)	867 (66.4)	Reference		Reference	
Malnutrition is causal or significant condition				.923		.256
Yes	37 (36.3)	480 (36.8)	0.98 (.64–1.48)		1.33 (.81–2.16)	
No	65 (63.7)	826 (63.2)	Reference		Reference	

Data are presented as No. (%) unless otherwise indicated.

Abbreviations: CI, confidence interval; CMV, cytomegalovirus; HIV, human immunodeficiency virus; OR, odds ratio; SD, standard deviation.

^a^Adjusted for all other variables listed in the model.

## DISCUSSION

Our analysis of nearly 6000 under-5 deaths and stillbirths in 7 LMICs found CMV infection in 19.5% of cases, with 2.4% having CMV disease as a causal factor leading to death. Prevalence of CMV infection and disease varied by country, being lower in Bangladesh than in African countries. CMV disease was more common in early infants (13.1%) and late infants (9.7%) than in stillbirths, neonates, or children (0.8%–3.3%). Among deaths with CMV disease as a causal factor, 100% of stillbirths and 64.7% of early neonatal cases had CMV as an underlying cause, compared to <30% (15.2%–29.4%) in other age groups. Of 139 under-5 deaths attributed to CMV disease, 45 (32.4%) were related to cCMV, with the remainder likely involving either congenital or postnatal infections. Lung involvement, often presenting as viral pneumonitis, was the most common pathology. Excluding HIV-related deaths, 76.9%, 46.2%, and 53.8% of infants with CMV disease in the causal pathway were severely underweight, stunted, and wasted, respectively.

Our findings showed a higher CMV infection prevalence among deaths in late infants and childhood compared with stillbirths, neonates, and early infants, suggesting postnatal acquisition in addition to cCMV or reactivation of dormant virus. Postnatal CMV infection is commonly transmitted via breastmilk (40%–65%) [4, 5]. While breastfeeding data were not collected in this study, higher breastfeeding rates in LMICs may explain the elevated infection rates, along with potential transmission from close household contacts. In utero infection and reactivation due to immunosuppression remain possible, particularly in neonates and infants, where cCMV likely contributes significantly. In high-income countries, infant deaths occur in 3%–10% of symptomatic cCMV cases or 0.3%–1% of all infants with cCMV [9].

CMV disease in stillbirths was lower (0.9% vs 4%) but similar for neonates (0.8% vs 1%) in this study compared to a previous study from the same sites [19], likely due to larger sample size in this study (N = 2204 vs N = 180). Among deaths beyond neonatal age, 7.2% were attributed to CMV disease, lower than the 11.9% reported in pneumonia deaths and 12% in infectious disease deaths in prior CHAMPS analyses [19, 28]. In early infants and late infants (28 days–12 months), the proportion was 10.9%, consistent with a South African study attributing 10.4% (7/67) of infant deaths to CMV, though that study reported no CMV-related deaths in children aged >12 months, compared to 3.3% in this study [29]

Approximately 30% of children in this study were HIV infected. Immunosuppression, a hallmark of HIV infection, not only increases CMV infection risk but also raises the possibility of a 2-way relationship. Previous research suggests that CMV infection itself could increase HIV acquisition risk in children breastfed by infected mothers [30–32]. Studies from Africa reported higher cCMV infection prevalence in HIV-exposed neonates compared with HIV-unexposed neonates [33, 34]. Similarly, other studies have shown a greater CMV infection risk during early childhood among children with HIV, particularly in the first year of life [30–32, 35]. Coinfection with HIV and CMV accelerates HIV disease progression, with many children dying by 18 months. This relationship could explain the relative lower CMV infection rates in Bangladesh and Ethiopia compared to other countries as they both had no HIV-exposed or infected deaths. Mortality in infants with cCMV has been reported to be higher when coinfected with HIV [36].

The proportion of deaths with microcephaly was higher among those with CMV disease in the causal pathway, with approximately 60% of such deaths showing microcephaly. This is much higher than reported in general populations in Uganda and South Africa [37, 38] but aligns with findings in neonates with symptomatic cCMV [39]. Congenital infections, including CMV, are major risk factors for microcephaly [40], suggesting that a significant proportion of these deaths were related to cCMV.

Study limitations include difficulties determining as to what proportion of CMV deaths were due to cCMV, compared to postnatally acquired CMV, even though the DeCoDe process considered premortem clinical signs typical of cCMV. Furthermore, our analysis is limited by the inability to calculate absolute death rates from CMV due to the lack of birth cohort data, which restricts assessment of the true population-level burden of CMV-related mortality. Histopathological examination was not performed on all organs, including placentas in stillbirths, potentially underestimating the impact of CMV disease on mortality. Limited information on antiretroviral therapy use in mothers and children and CMV treatment restricts assessment of their impact on mortality.

## CONCLUSIONS

Our analysis underscores the substantial role of CMV disease in under-5 child mortality. The high prevalence of microcephaly suggests cCMV plays a significant role. While antivirals for CMV infections might not be readily available in many LMICs, and cannot prevent in utero acquisition of infection, maternal vaccination and improved identification and treatment of HIV-infected children could help reduce CMV-related deaths.

## Supplementary Material

ciaf098_Supplementary_Data

## Supplementary Data

Supplementary materials are available at *Clinical Infectious Diseases* online. Consisting of data provided by the authors to benefit the reader, the posted materials are not copyedited and are the sole responsibility of the authors, so questions or comments should be addressed to the corresponding author.

## Notes

***Author Contributions.*** Q. B., S. O. S., S. E. A., S. A. M., D. O., J. A. G. S., D. M. B., I. M., E. S. G., V. A., K. L. K., M. D. T., and N. A. designed the protocol and led the involvement of sites in the CHAMPS network. S. V., B. T.-B., E. A. R., S. G. L., S. M., P. J. S., V. A., D. O., K. O., J. A. W., Q. B., C. C., I. M., D. T.-F., R. V., R. L., F. M., P. N.-P., I. U. O., J. O., S. E. A., E. S. G., N. A., L. G., L. M., J. A. G. S., H. W., J. J., A. M. K., K. L. K., S. O. S., M. D. T., P. M., and S. A. M., coordinated the implementation of CHAMPS procedures in the sites and the determination of causes of death for all cases. S. V., S. A. M., Z. J. M., D. M. B., B. T.-B., and C. G. W. conceptualized the manuscript. Z. J. M. directed data management and conducted data analysis. Z. J. M. and D. M. B. directly accessed and verified the underlying data reported in the manuscript. S. V. drafted the manuscript. All authors revised the manuscript. S. V., Z. J. M., D. M. B., C. G. W., and S. A. M. had final responsibility for the decision to submit the manuscript for publication. All authors reviewed and provided inputs the draft and approved the decision to submit for publication.

***Acknowledgments.*** CHAMPS would like to extend its deepest appreciation to all the families who participated. The network would like to acknowledge members who comprise the minimally invasive tissue sampling, Demographic Surveillance System, Social Behavioral Science, Information Technology, and laboratory teams and local communities across all 7 sites: Sazzad Hossain, Saiful Islam, Abu Faisal Md Pervez, and Muhammad Faruqe Hussain of CHAMPS Bangladesh; Yadeta Dessie, Tseyon Tesfaye, Haleluya Leulseged, Tadesse Dufera, Hiwot Yigzaw, Samuel Tigistu, Eyoel Taye, Mahlet Mekonnen, Andualem Alemayehu, Mussie Brhane, Mulu Berihun, Ketema Degefa, Berhanu Damisse, Zerihun Girma Gudata, Merga Deresa, Dadi Marami, Zelalem Teklemariam Kidanemariam, and Markus Brienes of CHAMPS Ethiopia; Janet Agaya, Bob Awino, Dickson Gethi, Elizabeth Oele, Richared Oliech, Fredrick Omondi, Clayton Onyango, Peter Onyango Nyamthimba, Louis Othieno, Kephas Otieno, Peter Otieno, and Nixon Ouma of CHAMPS Kenya; Mahamane Djiteye, Kounandji Diarra, Tieman Diarra, Issa Fofana, Rokiatou dite Foune Sacko, Yacouba Dogoni, Baba Seydou Kante, and Boubou Tamboura of CHAMPS Mali; Jeffrey B. Swims of the US Centers for Disease Control and Prevention (CDC); and Nellie Myburgh, Cleopas Hwinya, and Ismail Shiraz of CHAMPS South Africa.

***Data availability.*** CHAMPS data are open access and datasets are available for download or request at www.champshealth.org.

***Disclaimer.*** The funder participated in discussions of study design and data collection but did not participate in the conduct of the study; the management, analysis, or interpretation of the data; preparation, review, or approval of the manuscript; or decision to submit the manuscript for publication. Several authors are employed by the CDC. The findings and conclusions in this report are those of the author(s) and do not necessarily represent the official position of the CDC.

***Financial support.*** This work was supported by the Gates Foundation (grant number OPP1126780).
