# Supplementary material for: Investigating the Role of Cytomegalovirus as a Cause of Stillbirths and Child Deaths in Low- and Middle-Income Countries Through Postmortem Minimally Invasive Tissue Sampling
Source: Clin Infect Dis. 2025 Mar 10;82(2):326–36. doi: 10.1093/cid/ciaf098 (PMC13017237; doi:10.1093/cid/ciaf098)
Supplement: ciaf098_Supplementary_Data [file ciaf098_supplementary_data.docx]

**Supplementary Material**

| **Table S1.**  Frequencies of CMV infection and disease among all deaths by site, CHAMPS, December 2016 – July 2023. | | | | | | | |
| --- | --- | --- | --- | --- | --- | --- | --- |
| **Classification** | **Bangladesh**  N = 705  n/N (%) | **Ethiopia**  N = 837  n/N (%) | **Kenya**  N = 776  n/N (%) | **Mali**  N = 385  n/N (%) | **Mozambique**  N = 1216  n/N (%) | **Sierra Leone**  N = 775  n/N (%) | **South Africa**  N = 1147  n/N (%) |
| **CMV infection** | **21/705 (3.0)** | **52/837 (6.2)** | **264/776 (34.0)** | **56/385 (14.5)** | **231/1216 (19.0)** | **305/775 (39.4)** | **211/1147 (18.4)** |
| Stillbirth | 10/358 (2.8) | 14/511 (2.7) | 15/223 (6.7) | 3/142 (2.1) | 31/465 (6.7) | 19/214 (8.9) | 19/291 (6.5) |
| Neonate | 8/337 (2.4) | 7/250 (2.8) | 9/223 (4.0) | 9/154 (5.8) | 27/509 (5.3) | 35/226 (15.5) | 44/530 (8.3) |
| Early infant | 1/5 (20.0) | 1/14 (7.1) | 21/39 (53.8) | 8/22 (36.4) | 15/34 (44.1) | 19/38 (50.0) | 42/108 (38.9) |
| Late infant | 1/2 (50.0) | 8/22 (36.4) | 112/141 (79.4) | 15/30 (50.0) | 51/68 (75.0) | 79/98 (80.6) | 57/113 (50.4) |
| Child | 1/3 (33.3) | 22/40 (55.0) | 107/150 (71.3) | 21/37 (56.8) | 107/140 (76.4) | 153/199 (76.9) | 49/105 (46.7) |
| **CMV disease** | **12/705 (1.7)** | **23/837 (2.7)** | **63 (8.1)** | **19/385 (4.9)** | **105/1216 (8.6)** | **127/775 (16.4)** | **101/1147 (8.8)** |
| Stillbirth | 4/358 (1.1) | 9/511 (1.8) | 3/223 (1.3) | 1/142 (0.7) | 16/465 (3.4) | 2/214 (0.9) | 6/291 (2.1) |
| Neonate | 5/337 (1.5) | 2/250 (0.8) | 3/223 (1.3) | 3/154 (1.9) | 8/509 (1.6) | 7/226 (3.1) | 20/530 (3.8) |
| Early infant | 1/5 (20.0) | 1/14 (7.1) | 10/39 (25.6) | 2/22 (9.1) | 6/34 (17.6) | 12/38 (31.6) | 29/108 (26.9) |
| Late infant | 1/2 (50.0) | 6/22 (27.3) | 31/141 (22.0) | 9/30 (30.0) | 24/68 (35.3) | 45/98 (45.9) | 35/113 (31.0) |
| Child | 1/3 (33.3) | 5/40 (12.5) | 16/150 (10.7) | 4/37 (10.8) | 51/140 (36.4) | 61/199 (30.7) | 11/105 (10.5) |
| **CMV disease in causal pathway** | **7/705 (1.0)** | **5/837 (0.6)** | **24/776 (3.1)** | **5/385 (1.3)** | **22/1216 (1.8)** | **22/775 (2.8)** | **54/1147 (4.7)** |
| Stillbirth | 4/358 (1.1) | 2/511 (0.4) | 1/223 (0.4) | 0/142 (0.0) | 7/465 (1.5) | 0/214 (0.0) | 6/291 (2.1) |
| Neonate | 2/337 (0.6) | 0/250 (0.0) | 2/223 (0.9) | 0/154 (0.0) | 1/509 (0.2) | 3/226 (1.3) | 9/530 (1.7) |
| Early infant | 1/5 (20.0) | 1/14 (7.1) | 7/39 (17.9) | 1/22 (4.5) | 1/34 (2.9) | 3/38 (7.9) | 20/108 (18.5) |
| Late infant | 0/2 (0.0) | 0/22 (0.0) | 11/141 (7.8) | 3/30 (10.0) | 6/68 (8.8) | 9/98 (9.2) | 17/113 (15.0) |
| Child | 0/3 (0.0) | 2/40 (5.0) | 3/150 (2.0) | 1/37 (2.7) | 7/140 (5.0) | 7/199 (3.5) | 2/105 (1.9) |
| **CMV is immediate cause of death** | **0/705 (0.0)** | **2/837 (0.2)** | **16/776 (2.1)** | **1/385 (0.3)** | **7/1216 (0.6)** | **9/775 (1.2)** | **24/1147 (2.1)** |
| Stillbirth | 0/358 (0.0) | 0/511 (0.0) | 0/223 (0.0) | 0/142 (0.0) | 0/465 (0.0) | 0/214 (0.0) | 0/291 (0.0) |
| Neonate | 0/337 (0.0) | 0/250 (0.0) | 0/223 (0.0) | 0/154 (0.0) | 0/509 (0.0) | 1/226 (0.4) | 0/530 (0.0) |
| Early infant | 0/5 (0/0) | 1/14 (7.1) | 5/39 (12.8) | 1/22 (4.5) | 1/34 (2.9) | 2/38 (5.3) | 12/108 (11.1) |
| Late infant | 0/2 (0.0) | 0/22 (0.0) | 10/141 (7.1) | 0/30 (0.0) | 2/68 (2.9) | 4/98 (4.1) | 11/113 (9.7) |
| Child | 0/3 (0.0) | 1/40 (2.5) | 1/150 (0.7) | 0/37 (0.0) | 4/140 (2.9) | 2/199 (1.0) | 1/105 (1.0) |
| **CMV is antecedent cause of death** | **2/705 (0.3)** | **3/837 (0.4)** | **7/776 (0.9)** | **4/385 (1.0)** | **7/1216 (0.6)** | **6/775 (0.8)** | **21/1147 (1.8)** |
| Stillbirth | 0/358 (0.0) | 0/511 (0.0) | 0/223 (0.0) | 0/142 (0.0) | 0/465 (0.0) | 0/214 (0.0) | 0/291 (0.0) |
| Neonate | 2/337 (0.6) | 0/250 (0.0) | 1/223 (0.4) | 0/154 (0.0) | 0/509 (0.0) | 1/226 (0.4) | 2/530 (0.4) |
| Early infant | 0/5 (0/0) | 1/14 (7.1) | 4/39 (10.3) | 0/22 (0.0) | 0/34 (0.0) | 1/38 (2.6) | 11/108 (10.2) |
| Late infant | 0/2 (0.0) | 0/22 (0.0) | 1/141 (0.7) | 3/30 (10.0) | 4/68 (5.9) | 2/98 (2.0) | 7/113 (6.2) |
| Child | 0/3 (0.0) | 2/40 (5.0) | 1/150 (0.7) | 1/37 (2.7) | 3/140 (2.1) | 2/199 (1.0) | 1/105 (1.0) |
| **CMV is underlying cause of death** | **5/705 (0.7)** | **3/837 (0.4)** | **4/776 (0.5)** | **0/385 (0.0)** | **9/1216 (0.7)** | **9/775 (1.2)** | **24/1147 (2.1)** |
| Stillbirth | 4/358 (1.1) | 2/511 (0.4) | 1/223 (0.4) | 0/142 (0.0) | 7/465 (1.5) | 0/214 (0.0) | 6/291 (2.1) |
| Neonate | 0/337 (0.0) | 0/250 (0.0) | 1/223 (0.4) | 0/154 (0.0) | 1/509 (0.2) | 1/226 (0.4) | 8/530 (1.5) |
| Early infant | 1/5 (20.0) | 1/14 (7.1) | 1/39 (2.6) | 0/22 (0.0) | 0/34 (0.0) | 2/38 (5.3) | 5/108 (4.6) |
| Late infant | 0/2 (0.0) | 0/22 (0.0) | 0/141 (0.0) | 0/30 (0.0) | 0/68 (0.0) | 3/98 (3.1) | 4/113 (3.5) |
| Child | 0/3 (0.0) | 0/40 (0.0) | 1/150 (0.7) | 0/37 (0.0) | 1/140 (0.7) | 3/199 (1.5) | 1/105 (1.0) |
| **CMV**: cytomegalovirus  **CMV infection**: TAC PCR positive on any specimen  **CMV disease**: presence of histopathologic changes on any specimen OR disseminated CMV defined as positive CMV PCR in ≥2 specimens (considering NP/OP swabs and lung tissues as one specimen)  **CMV in the causal pathway**: CMV is the immediate, antecedent, or underlying cause of death. Some deaths may have CMV multiple times in the causal chain. | | | | | | | |

| **Table S2**: Distribution of cycle threshold values for samples testing positive for cytomegalovirus (CMV) on the TaqMan Array Card nucleic acid amplification assay by whether CMV was attributed in the etiology of death or not implicated in the causal pathway, CHAMPS, December 2016 – July 2023. | | | | | | |
| --- | --- | --- | --- | --- | --- | --- |
| Number PCR  Positive | Minimum | Lower quartile | Median | Mean | Upper quartile | Maximum |
| **CMV in the causal chain** (N=137) | | | | | | |
| Tissue specimen from lung 126 | 15.01 | 23.77 | 25.97 | 26.38 | 29.26 | 39.24 |
| Nasopharyngeal swab 91 | 15.80 | 23.57 | 25.97 | 26.56 | 30.23 | 33.75 |
| Whole blood 76 | 17.97 | 26.74 | 29.94 | 29.27 | 32.01 | 39.24 |
| Cerebrospinal fluid sample 47 | 23.81 | 29.05 | 31.00 | 30.92 | 33.64 | 36.72 |
| **CMV disease, but not in the causal chain** (N=308) | | | | | | |
| Tissue specimen from lung 232 | 17.43 | 27.35 | 30.68 | 30.14 | 33.19 | 43.05 |
| Nasopharyngeal swab 224 | 15.26 | 25.42 | 29.18 | 28.82 | 32.39 | 42.47 |
| Whole blood 272 | 15.29 | 29.90 | 32.41 | 31.93 | 34.29 | 44.63 |
| Cerebrospinal fluid sample 79 | 26.55 | 32.04 | 33.71 | 33.86 | 35.76 | 39.91 |
| **CMV infection without CMV disease** (N=690) | | | | | | |
| Tissue specimen from lung 330 | 21.44 | 29.55 | 31.95 | 31.76 | 34.33 | 41.04 |
| Nasopharyngeal swab 472 | 18.64 | 26.81 | 30.25 | 29.81 | 32.75 | 42.33 |
| Whole blood 84 | 18.75 | 31.20 | 32.65 | 32.66 | 35.25 | 41.10 |
| Cerebrospinal fluid sample 21 | 27.12 | 30.45 | 35.42 | 33.90 | 36.23 | 40.61 |
| **CMV**: cytomegalovirus,  Ct values for similar specimens from decedents with CMV disease in the causal chain were significantly lower than those in CMV disease not in causal chain or infected (p<0.001) (Kruskal Wallis Test) | | | | | | |

| **Table S3.**  Characteristics of deaths with CMV disease in causal pathway by site, CHAMPS, December 2016 – July 2023. | | | | | | | |
| --- | --- | --- | --- | --- | --- | --- | --- |
|  | **Bangladesh**  N = 7  n (%) | **Ethiopia**  N = 5  n (%) | **Kenya**  N = 24  n (%) | **Mali**  N = 25  n (%) | **Mozambique**  N = 22  n (%) | **Sierra Leone**  N = 22  n (%) | **South Africa**  N = 54  n (%) |
| **Age group** (%) |  |  |  |  |  |  |  |
| Stillbirth | 4 (57.1) | 2 (40.0) | 1 (4.2) | 0 (0.0) | 7 (31.8) | 0 (0.0) | 6 (11.1) |
| Neonate | 2 (28.6) | 0 (0.0) | 2 (8.3) | 0 (0.0) | 1 (4.5) | 3 (13.6) | 9 (16.7) |
| Early infant | 1 (14.3) | 1 (20.0) | 7 (29.2) | 1 (20.0) | 1 (4.5) | 3 (13.6) | 20 (37.0) |
| Late infant | 0 (0.0) | 0 (0.0) | 11 (45.8) | 3 (60.0) | 6 (27.3) | 9 (40.9) | 17 (31.5) |
| Child | 0 (0.0) | 2 (40.0) | 3 (12.5) | 1 (20.0) | 7 (31.8) | 7 (31.8) | 2 ( 3.7) |
| **Sex** (%) |  |  |  |  |  |  |  |
| Female | 3 (42.9) | 2 (40.0) | 8 (33.3) | 3 (60.0) | 10 (45.5) | 11 (50.0) | 22 (40.7) |
| Male | 4 (57.1) | 3 (60.0) | 16 (66.7) | 2 (40.0) | 12 (54.5) | 11 (50.0) | 32 (59.3) |
| **Location of death** (%) |  |  |  |  |  |  |  |
| Facility | 7 (100.0) | 2 (40.0) | 14 (58.3) | 4 (80.0) | 22 (100.0) | 20 (90.9) | 51 (94.4) |
| Community | 0 (0.0) | 3 (60.0) | 10 (41.7) | 1 (20.0) | 0 (0.0) | 2 (9.1) | 3 (5.6) |
| **Hospital duration in hours** (median [IQR]) (N=71) | 7 [5, 19] | 69 [69, 69] | 30 [24, 72] | 87 [60, 113] | 95 [8, 147] | 29 [10, 64] | 171 [50, 1200] |
| **Time from death to MITS in hours** (median [IQR]) (N=101) | 1 [1, 2] | 5 [2, 6] | 18 [9, 26] | 7 [4, 13] | 4 [2, 15] | 4 [2, 9] | 27 [17, 42] |
| **HIV status** (%) |  |  |  |  |  |  |  |
| Uninfected or unknown | 7 (100.0) | 5 (100.0) | 11 (45.8) | 1 (20.0) | 8 (36.4) | 19 (86.4) | 21 (38.9) |
| Exposed uninfected | 0 (0.0) | 0 (0.0) | 4 (16.7) | 0 (0.0) | 5 (22.7) | 0 (0.0) | 19 (35.2) |
| Infected | 0 (0.0) | 0 (0.0) | 9 (37.5) | 4 (80.0) | 9 (40.9) | 3 (13.6) | 14 (25.9) |
| **Weight-for-age Z-score** (N=65)^a^ |  |  |  |  |  |  |  |
| Normal (≥2SD) | 0 (0.0) | 0 (0.0) | 1 (7.7) | 0 (0.0) | 1 (20.0) | 4 (25.0) | 4 (15.4) |
| Moderate underweight (<-2SD to -3SD) | 0 (0.0) | 0 (0.0) | 0 (0.0) | 0 (0.0) | 1 (20.0) | 3 (18.8) | 1 (3.8) |
| Severe underweight (<-3SD) | 1 (100.0) | 3 (100.0) | 12 (92.3) | 1 (100.0) | 3 (60.0) | 9 (56.2) | 21 (80.8) |
| **Height-for-age Z-score** (N=65)^a^ |  |  |  |  |  |  |  |
| Normal (≥2SD) | 1 (100.0) | 0 (0.0) | 4 (30.8) | 0 (0.0) | 2 (40.0) | 9 (56.2) | 6 (23.1) |
| Moderate stunting (<-2SD to -3SD) | 0 (0.0) | 0 (0.0) | 4 (30.8) | 0 (0.0) | 2 (40.0) | 3 (18.8) | 4 (15.4) |
| Severe stunting (<-3SD) | 0 (0.0) | 3 (100.0) | 5 (38.5) | 1 (100.0) | 1 (20.0) | 4 (25.0) | 16 (61.5) |
| **Weight-for-height Z-score** (N=52)^a^ |  |  |  |  |  |  |  |
| Normal (≥2SD) | 0 (0.0) | 2 (100.0) | 2 (16.7) | 0 (0.0) | 1 (20.0) | 3 (20.0) | 6 (37.5) |
| Moderate wasting (<-2SD to -3SD) | 0 (0.0) | 0 (0.0) | 0 (0.0) | 0 (0.0) | 4 (80.0) | 3 (20.0) | 3 (18.8) |
| Severe wasting (<-3SD) | 1 (100.0) | 0 (0.0) | 10 (83.3) | 1 (100.0) | 0 (0.0) | 9 (60.0) | 7 (43.8) |
| **Mid-upper arm circumference (cm) Z-score** (N=38)^a^ |  |  |  |  |  |  |  |
| Normal (≥2SD) | – | 1 (50.0) | 1 (12.5) | 0 (0.0) | 2 (50.0) | 7 (53.8) | 4 (40.0) |
| Moderate malnutrition (<-2SD to -3SD) | – | 0 (0.0) | 1 (12.5) | 0 (0.0) | 1 (25.0) | 2 (15.4) | 1 (10.0) |
| Severe malnutrition (<-3SD) | – | 1 (50.0) | 6 (75.0) | 1 (100.0) | 1 (25.0) | 4 (30.8) | 5 (50.0) |
| **Head circumference Z-score** |  |  |  |  |  |  |  |
| Normal (≥2SD) | 0 (0.0) | 1 (33.3) | 9 (39.1) | 2 (40.0) | 7 (46.7) | 12 (54.5) | 17 (35.4) |
| Moderate microcephaly (<-2SD to -3SD) | 1 (33.3) | 0 (0.0) | 6 (26.1) | 1 (20.0) | 4 (26.7) | 3 (13.6) | 4 (8.3) |
| Severe microcephaly (<-3SD) | 2 (66.7) | 2 (66.7) | 8 (34.8) | 2 (40.0) | 4 (26.7) | 7 (31.8) | 27 (56.2) |
| **Birth weight** (%) |  |  |  |  |  |  |  |
| Extremely low birth weight | 2 (28.6) | 0 (0.0) | 1 (4.2) | 0 (0.0) | 0 (0.0) | 0 (0.0) | 6 (11.1) |
| Very low birth weight | 2 (28.6) | 0 (0.0) | 1 (4.2) | 0 (0.0) | 1 (4.5) | 1 (4.5) | 9 (16.7) |
| Low birth weight | 1 (14.3) | 0 (0.0) | 5 (20.8) | 0 (0.0) | 4 (18.2) | 1 (4.5) | 13 (24.1) |
| Normal weight | 0 (0.0) | 0 (0.0) | 10 (41.7) | 2 (40.0) | 8 (36.4) | 10 (45.5) | 4 (7.4) |
| Missing | 2 (28.6) | 5 (100.0) | 7 (29.2) | 3 (60.0) | 9 (40.9) | 10 (45.5) | 22 (40.7) |
| **Count of causal conditions identified** (%) |  |  |  |  |  |  |  |
| 1 | 2 (28.6) | 0 (0.0) | 2 (8.3) | 0 (0.0) | 7 (31.8) | 3 (13.6) | 10 (18.5) |
| 2 | 2 (28.6) | 1 (20.0) | 11 (45.8) | 1 (20.0) | 5 (22.7) | 4 (18.2) | 9 (16.7) |
| 3 | 3 (42.9) | 1 (20.0) | 8 (33.3) | 0 (0.0) | 2 (9.1) | 5 (22.7) | 11 (20.4) |
| ≥4 | 0 (0.0) | 3 (60.0) | 3 (12.5) | 4 (80.0) | 8 (36.4) | 10 (45.5) | 24 (44.4) |
| Median [IQR] | 2 [1, 3] | 4 [3, 4] | 2 [2, 3] | 5 [4, 5] | 2 [1, 4] | 3 [2, 4] | 3 [2, 4] |
| **Deemed preventable or preventable under certain conditions from DeCoDe panel** (%) | 6 (85.7) | 5 (100.0) | 23 (95.8) | 3 (60.0) | 13 (59.1) | 20 (90.9) | 31 (57.4) |
| **CMV:** cytomegalovirus; **SB–EN**: stillbirth or early neonate; **IQR**: interquartile range; **SD**: standard deviation; **DeCoDe**: Determination of Cause of Death panel.  ^a^ Excludes deaths with HIV as underlying cause and stillbirths/deaths <15 days. | | | | | | | |

| **Table S4.**  Associations between characteristics and CMV in the causal chain among deaths aged ≥28 days, CHAMPS, December 2016 – July 2023 (N = 618). | | | | | | |
| --- | --- | --- | --- | --- | --- | --- |
|  | **CMV in causal pathway**  N = 103  n (%) | **Deaths without CMV disease nor infection^b^**  N = 650  n (%) | **Crude Odds**  **Ratio**  **(95%CI)** | **P-value** | **Adjusted^a^ Odds Ratio**  **(95% CI)** | **P-value** |
| **Age group** |  |  |  | <0.001 |  | 0.009 |
| Early infant | 35 (34.0) | 287 (44.2) | 2.15 (1.22, 3.87) |  | 1.95 (0.96, 4.06) |  |
| Late infant | 46 (44.7) | 150 (23.1) | 2.97 (1.73, 5.22) |  | 2.75 (1.43, 5.45) |  |
| Child | 22 (21.4) | 213 (32.8) | *Reference* |  | *Reference* |  |
| **Sex** |  |  |  | 0.309 |  | 0.304 |
| Male | 63 (61.2) | 366 (56.4) | 1.25 (0.81, 1.94) |  | 1.31 (0.78, 2.21) |  |
| Female | 40 (38.8) | 283 (43.6) | *Reference* |  | *Reference* |  |
| **Location of death** |  |  |  | 0.012 |  | 0.080 |
| Facility | 86 (83.5) | 481 (74.6) | 1.96 (1.15, 3.52) |  | 1.84 (0.93, 3.87) |  |
| Community | 17 (16.5) | 164 (25.4) | *Reference* |  | *Reference* |  |
| **Site** |  |  |  | 0.270 |  | 0.204 |
| Bangladesh | 1 (1.0) | 18 (2.8) | 0.65 (0.03, 3.79) |  | 1.02 (0.05, 6.46) |  |
| Ethiopia | 3 (2.9) | 55 (8.5) | 0.30 (0.07, 0.88) |  | 0.40 (0.09, 1.39) |  |
| Kenya | 21 (20.4) | 107 (16.5) | 1.06 (0.58, 1.89) |  | 1.40 (0.67, 2.87) |  |
| Mali | 5 (4.9) | 52 (8.0) | 0.50 (0.17, 1.25) |  | 0.43 (0.11, 1.33) |  |
| Mozambique | 14 (13.6) | 101 (15.5) | 0.92 (0.46, 1.77) |  | 0.72 (0.30, 1.66) |  |
| Sierra Leone | 19 (18.4) | 94 (14.5) | 1.04 (0.56, 1.89) |  | 1.43 (0.67, 2.97) |  |
| South Africa | 40 (38.8) | 223 (34.3) | *Reference* |  | *Reference* |  |
| **HIV status** |  |  |  | <0.001 |  | <0.001 |
| Infected | 38 (36.9) | 13 (2.0) | 24.94 (12.74, 52.14) |  | 35.07 (16.49, 80.51) |  |
| Uninfected | 65 (63.1) | 637 (98.0) | *Reference* |  | *Reference* |  |
| **Head circumference Z-score** |  |  |  | 0.020 |  | 0.019 |
| Severe microcephaly (<-3SD) | 46 (45.1) | 310 (60.1) | 1.89 (1.18, 3.03) |  | 2.42 (1.31, 4.52) |  |
| Moderate microcephaly (<-2SD to -3SD) | 17 (16.7) | 67 (13.0) | 1.71 (0.90, 3.12) |  | 1.46 (0.65, 3.13) |  |
| Normal (≥2SD) | 39 (38.2) | 139 (26.9) | *Reference* |  | *Reference* |  |
| **Malnutrition is causal or significant condition** |  |  |  | 0.542 |  | 0.268 |
| Yes | 37 (35.9) | 178 (27.4) | 1.15 (0.73, 1.78) |  | 1.39 (0.77, 2.50) |  |
| No | 66 (64.1) | 472 (72.6) | *Reference* |  | *Reference* |  |
| **CMV:** cytomegalovirus; **CI**: confidence interval  **^a^** Adjusted for all other variables listed in the model.  ^b^ This table is the same as Table 4 but excludes deaths with CMV infection or CMV disease from the reference group. | | | | | | |

**Figure S1.** Other pathogens in deaths with CMV in the causal chain by age group, CHAMPS, December 2016 – July 2023

***
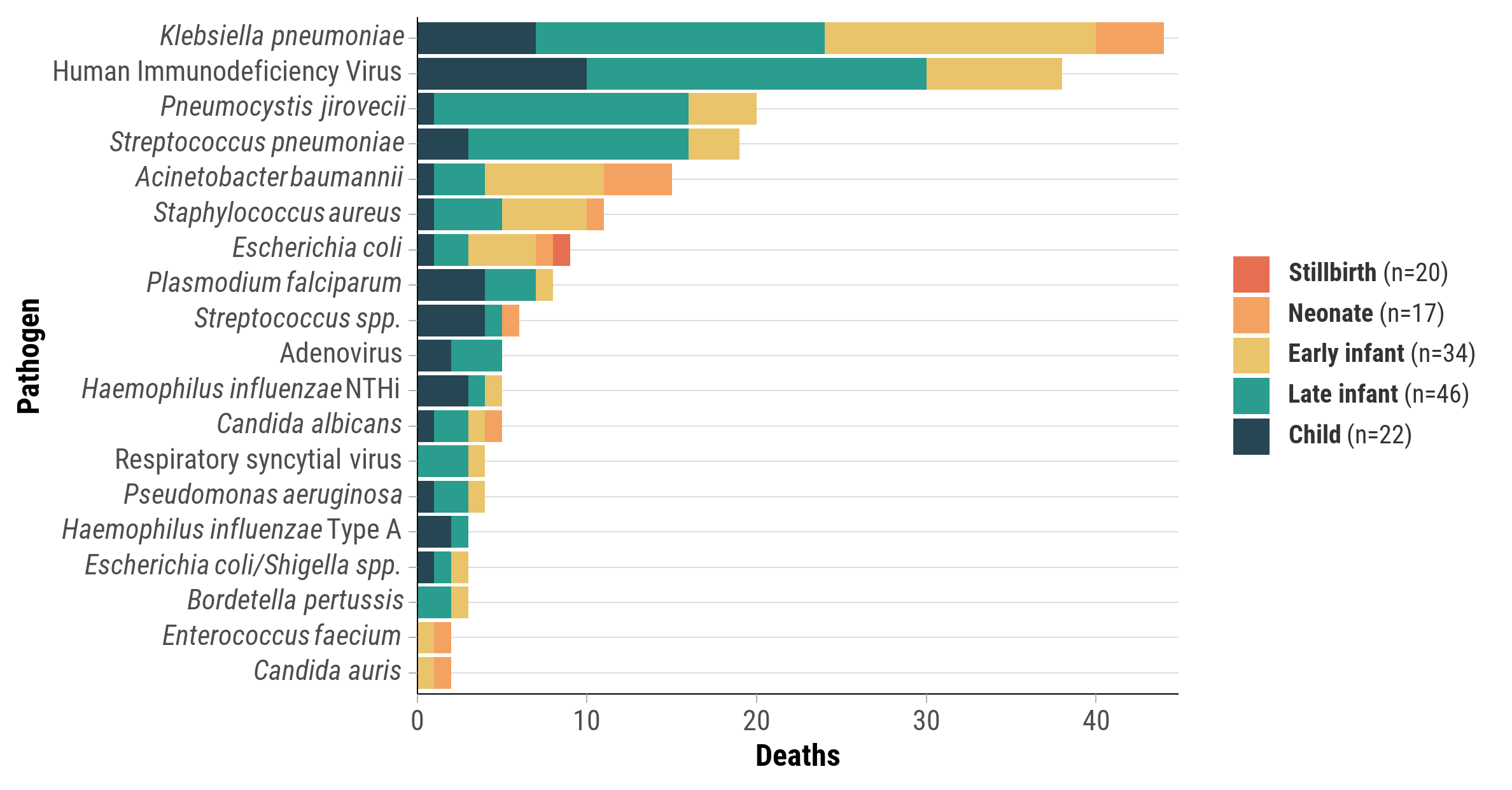
***

Pathogens implicated in at least two deaths are shown. Other pathogens implicated in one CMV death include *Bukholderia cepacea*, *Burkholderia pseudomallei*, *Candida parapsilosis*, *Enterobacter cloacae*, *Enterococcus spp.*, human metapneumovirus, influenza A, norovirus GI, parainfluenza virus type 3, parainfluenza virus type 4, *Proteus mirabilis*, rhinovirus, rotavirus A, *Salmonella spp.*, *Serratia marcescens*, *Stenotrophomonas maltophilia*, *Ureaplasma spp.*, and *Vibrio cholerae.*
